# Nature vs nurture of glucose homeostasis trajectories in children from the ALSPAC study

**DOI:** 10.1007/s00125-026-06722-5

**Published:** 2026-04-15

**Authors:** Isabel Gamache, Kaossarath Fagbemi, Soren Harnois-Leblanc, Celia M. T. Greenwood, Mélanie Henderson, Andrea Van Hulst, Nicholas J. Timpson, Despoina Manousaki

**Affiliations:** 1 Research Center, CHU Sainte-Justine, Montréal, Québec Canada; 2 Centre for Structural and Functional Genomics, Biology Department, Concordia University, Montréal, Québec Canada; 3 Department of Population Medicine, Harvard Pilgrim Health Care Institute and Harvard Medical School, Boston, MA USA; 4 Lady Davis Institute for Medical Research, Jewish General Hospital, Montréal, Québec Canada; 5 Gerald Bronfman Department of Oncology, McGill University, Montréal, Québec Canada; 6 Department of Epidemiology, Biostatistics and Occupational Health, McGill University, Montréal, Québec Canada; 7 Department of Paediatrics, Université de Montréal, Montréal, Québec Canada; 8 Department of Social and Preventive Medicine, School of Public Health, Université de Montréal, Montréal, Québec Canada; 9 Ingram School of Nursing, Faculty of Medicine and Health Science, McGill University, Montréal, Québec Canada; 10 Medical Research Council Integrative Epidemiology Unit, University of Bristol, Bristol, UK; 11 Population Health Sciences, Bristol Medical School, University of Bristol, Bristol, UK; 12 Department of Biochemistry and Molecular Medicine, Université de Montréal, Montréal, Québec Canada

**Keywords:** ALSPAC, Gene–environment interaction, Genetic analysis, Paediatric diabetes, Polygenic risk scores, Preventive genetics, Type 2 diabetes

## Abstract

**Aims/hypothesis:**

Dysglycaemia in youth results from complex interactions between genetic and environmental factors, yet their individual and combined contributions remain unclear. We aimed to: (1) evaluate the predictive performance of polygenic risk scores (PRSs) for glycaemic traits from childhood to early adulthood; (2) identify gene–environment interactions shaping glucose homeostasis trajectories; and (3) explore underlying mechanisms using pathway-specific PRSs.

**Methods:**

Data from 8783 participants (aged 7–24 years) from the Avon Longitudinal Study of Parents and Children (ALSPAC) were used to compute 12 PRSs for type 2 diabetes, fasting glucose, insulin and BMI. Glycaemic outcomes, including insulin resistance, prediabetes (impaired fasting glucose) and type 2 diabetes, were assessed using fasting glucose and insulin (ages 7, 15, 18 and 24 years) and HbA_1c_ (age 9 years). We evaluated whether PRSs could distinguish between transient (resolved by adulthood) and persistent glycaemic abnormalities using multinomial regression. We performed univariate and multivariate regressions, incorporating environmental factors (lifestyle, diet and maternal characteristics), and evaluated model performance using R^2^ and AUC. We tested interactions between PRS quintiles and environmental factors and explored pathophysiological mechanisms using pathway-specific PRSs.

**Results:**

Prediabetes prevalence was up to 24% at age 24 years. PRS-enhanced models outperformed those using environmental factors alone (e.g. AUC for dysglycaemia at age 15 improved by 0.12, reaching 0.78). Fasting glucose PRS showed moderate ability to differentiate transient from persistent prediabetes (AUC=0.70). Gene–environment analyses revealed that high genetic risk combined with increased screen time or younger maternal age increased insulin resistance risk. Physical activity and health awareness attenuated this risk. Pathway analyses indicated insulin secretion as a key mechanism earlier in life, with insulin resistance emerging later.

**Conclusions/interpretation:**

Genes interact with environment to define glucose homeostasis in youth, highlighting modifiable factors as actionable targets for early prevention.

**Graphical Abstract:**

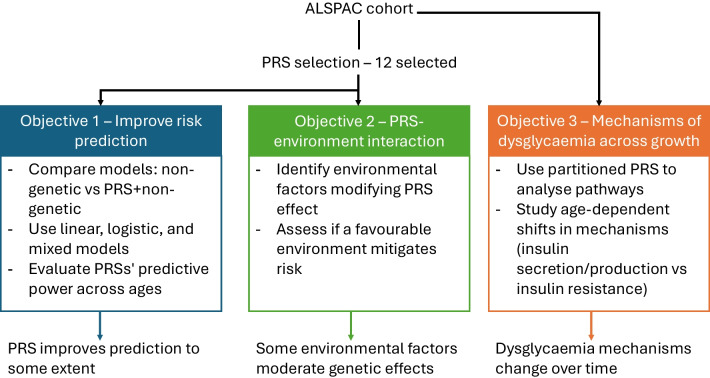

**Supplementary Information:**

The online version contains peer-reviewed but unedited supplementary material available at 10.1007/s00125-026-06722-5.


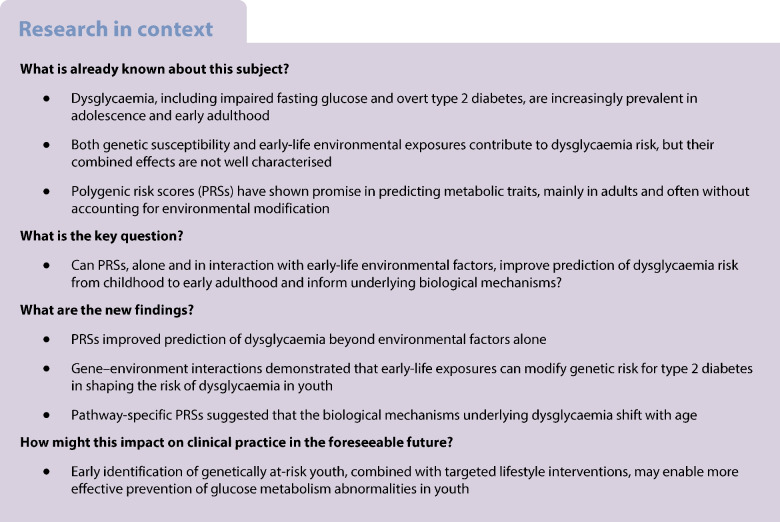


## Introduction

Over the past two decades, the incidence of youth-onset type 2 diabetes and prediabetes, which define an abnormal glucose metabolism, has surged worldwide affecting youth across all ancestries, including White Europeans [1]. Although more male than female individuals are likely to develop type 2 diabetes in adulthood, the opposite is true in early childhood [2, 3]. Moreover, paediatric type 2 diabetes exhibits distinct pathophysiological characteristics, higher rates of complications, and more limited treatment options compared with adult type 2 diabetes [4, 5]. In early stages, affected children display exaggerated insulin hypersecretion for a given level of insulin resistance, largely driven by excess adiposity, concurring with or followed by a decline in insulin secretion at a faster rate compared with that in adults [6, 7]. Previous studies have reported strong associations between youth dysglycaemia and obesity [8, 9], highlighting adiposity as a major determinant of insulin resistance during childhood and adolescence. Specific environmental factors contribute to the development of obesity and type 2 diabetes in youth, including a sedentary lifestyle, physical inactivity [10], and a nutrient-poor and high-energy diet relying on ultra-processed foods [11, 12]. However, not all children with obesity will progress from prediabetes to overt type 2 diabetes [13], highlighting the need for further investigation into the genetic and environmental factors shaping disease trajectories in youth, as well as their interactions. This knowledge could inform precision medicine strategies to prevent dysglycaemia and type 2 diabetes in youth.

The main goal of this project was to evaluate the predictive ability of type 2 diabetes polygenic risk scores (PRSs) from recent genome-wide association studies (GWASs) on glucose homeostasis trajectories and to investigate gene–environment interactions in youth of European ancestry from the Avon Longitudinal Study of Parents and Children (ALSPAC) [14–16]. The study had three objectives. First, to assess whether combining genetic and environmental factors (e.g. lifestyle, clinical, dietary and maternal) improves the prediction of dysglycaemia progression in children, compared with models using environmental data alone. We also examined whether PRSs could differentiate transient from persistent dysglycaemia. Second, we tested whether environment factors modify the effect of genetic susceptibility. Third, we investigated the pathophysiological mechanisms underlying glucose regulation in children using pathway-specific partitioned PRSs for adult type 2 diabetes [17]. For all these objectives, we explored potential sex-specific differences.

## Methods

### Description of the ALSPAC study

ALSPAC is a population-based, birth cohort study. Pregnant women residing in Avon, UK, with expected dates of delivery between 1 April 1991 and 31 December 1992 were invited to participate. Of 20,248 eligible pregnancies, 14,541 were initially enrolled, resulting in 13,988 children alive at 1 year. An additional 913 children were later enrolled, bringing the total sample for analyses after age 7 years to 15,447 pregnancies (14,833 unique women), with 14,901 children alive at 1 year. Mother’s partners were also invited to complete questionnaires, with 3807 currently enrolled. The study’s website provides a searchable data dictionary and variable search tool (https://www.bristol.ac.uk/alspac/researchers/our-data/). Over 28 years, 8932 offspring (43.9% female at birth) and their parents were repeatedly assessed via questionnaires and clinic-based evaluations. Full study details are available elsewhere [14–16]. At age 18 years, study children were sent ‘fair processing’ materials describing ALSPAC’s intended use of their health and administrative records and were given clear means to consent or object via a written form. Data were extracted for participants having provided informed consent. REDCap [18] electronic data capture tools are hosted at the University of Bristol. We used genetic data to define the sex of the participants.

#### Dysglycaemia-related phenotypes

Glucose-related traits, including fasting glucose (mmol/l) and insulin (µ/l) levels, were measured at four visits, at ages 7 (F7), 15 (F15), 18 (F18) and 24 (F24) years. HbA_1c_ levels were measured at age 9 years (F9). At F7, blood draws were performed before and 30 min after glucose load to obtain fasting and postprandial glucose (pg glucose) and insulin (pg insulin) levels to enable calculation of HOMA-IR. Outliers (>5SD from the mean) were excluded, and the remaining values were normalised using internal z-scores.

For the definition of diabetes and prediabetes, we used the Diabetes Canada criteria [19]. Diabetes was defined if fasting glucose exceeded 7 mmol/l, while prediabetes was defined as fasting glucose between 5.6 mmol/l and 7 mmol/l. At F7, HbA_1c_ levels from F9 were also considered: HbA_1c_ >6.5% (48 mmol/mol) indicated diabetes, while 6.0–6.5% (42–48 mmol/mol) indicated prediabetes. We defined prediabetes as meeting at least one of the two criteria, and required only one criterion to define diabetes, in contrast to the two criteria required by Diabetes Canada. At F18 and F24, physician-diagnosed or self-reported type 2 diabetes cases were added. The composite outcome of dysglycaemia included both definitions of type 2 diabetes and prediabetes at a given time point. Insulin resistance was defined by a HOMA-IR above the 2.5 cutoff used in previous studies [20]. As a sensitivity analysis, given transient variations in insulin resistance in adolescence, we also used full cohort sample-derived age-specific 85th percentile as a cutoff for high fasting glucose and HOMA-IR at each visit to define alternative dysglycaemia and insulin resistance outcomes, respectively. We excluded children diagnosed with type 1 diabetes, those undergoing insulin therapy, those taking diabetogenic antipsychotics, or girls who were pregnant at any visit.

#### Environmental factors

For environmental, maternal, lifestyle and socio-demographic risk factors, hereafter referred to as environmental factors, we selected a wide range of variables measured as prenatal exposures, at birth or during follow-up visits. These included breastfeeding duration, diet, physical activity and socioeconomic indicators. Electronic supplementary material (ESM) Table 1a shows the detailed list of variables and their definition in ALSPAC. We also considered maternal factors including pre-pregnancy anthropometric and socioeconomic variables, dietary habits and other environmental exposures during pregnancy (ESM Table 1b).

### PRS selection

Based on paediatric and adult GWAS data from MAGIC (Meta-Analyses of Glucose and Insulin-related traits Consortium; www.magicinvestigators.org), we derived 33 polygenic PRSs for various glucose homeostasis traits. Of these, 9 PRSs were previously published, while 24 were newly developed using a clumping and thresholding approach using PRSice2. We applied a more liberal *p* threshold of 1 × 10^−5^ to accommodate the reduced statistical power from smaller paediatric GWAS. Clumping was performed using an r^2^ threshold of 0.1 (ESM Table 2). No optimisation step was performed due to the lack of a validation cohort.

To identify the most predictive PRSs for the eight outcomes (prediabetes, type 2 diabetes, HbA_1c_, fasting glucose, fasting insulin, pg glucose, pg insulin, and HOMA-IR), we conducted univariate linear and logistic regression analyses. Predictive performance was assessed using AUC (via *ROCit* and *pROC* R packages) or R^2^ values. PRSs not associated with any of the outcomes (*p*>0.05) were excluded. We then identified PRSs that frequently ranked in the top 10 based on AUC or R^2^, selecting the most consistently top-performing PRS per phenotype category. This process led to the selection of 12 PRSs retained in further analyses (ESM Table 2).

To investigate the pathophysiological mechanisms underlying the progression of dysglycaemia in childhood, we employed published partitioned PRSs (pPRSs) for adult type 2 diabetes [17]. Specifically, five pPRSs correspond to two main mechanisms. The first mechanism involves insulin production and secretion and includes a beta cell function PRS (Beta Cell, N_SN*p*_=30) and a PRS associated with proinsulin production (Proinsulin, N_SN*p*_=7). The second mechanism relates to insulin resistance and consists of three pPRSs: one associated with obesity-related traits (Obesity, N_SN*p*_=5), another linked to abnormal fat distribution and metabolic changes (Lipodystrophy, N_SN*p*_=20) and a third related to liver function and lipid profiles (Liver-Lipid, N_SN*p*_=5).

### Cross-sectional analysis

We evaluated the improvement in outcome prediction performance gained by incorporating PRS into various models. Model 1 included a PRS, sex, age and the top five genotype-derived principal components (PCs) to account for population stratification and batch effect, which were quantified using flashPCA2. Model 2 included age, sex, PCs and all environmental factors associated with the outcome from Model 1 (at a Bonferroni-adjusted *p*). Model 3 incorporated a PRS into Model 2.

Binary outcomes with at least 100 cases were analysed, which represent a prevalence of about 5%, while continuous outcomes required a minimum of 500 observations at a given visit. Environmental factors measured at the same visit as the outcome or previous visits were considered as potential covariates.

To set the significance threshold for including environmental covariates in Models 2 and 3, we considered nine independent categories (child and maternal physical activity, child anthropometry, maternal pregnancy information, diet for both, socio-demographic factors for both, and maternal smoking), plus a genetic category, leading to a Bonferroni-corrected significance threshold of *p*<0.05/10=0.005. Given the strong representation of BMI-associated SNPs in glucose homeostasis-related PRSs, and the high correlation between adiposity measures and many PRSs (ESM Table 3), we did not adjust for any adiposity measures in any models to avoid collider bias [21].

To mitigate collinearity in Models 2 and 3, we identified significant environmental factors associated with the outcome by computing Pearson correlation coefficients between all environmental variables. Pairs with |r| >0.6 were flagged, and the variable with the least significant *p* of the pair (in Model 1) was removed. Second, we computed the variance inflation factor and excluded variables with a variance inflation factor >5.

All analyses were conducted in the entire cohort and in sex-stratified models. The gain in predictive performance following the addition of PRS was assessed by comparing the R^2^ or AUC between Model 2 and Model 3.

### Longitudinal analysis

We used linear mixed-effects models (LMMs) in the *nlme* R package to analyse associations between each PRS, pPRS, or suggestive environmental factors (*p*<0.05 in at least one visit in the univariate model [Model 1]) and continuous outcomes at all visits. Due to convergence error using LMM, binary outcomes were evaluated using the generalised estimating equation using *geepack* R package. The time variable was defined using the mean age at each visit, which we then transformed to have baseline age set at 0. Quadratic terms were tested to assess nonlinearity between mean age and continuous outcomes. We additionally tested for interactions between mean age and the PRS. To determine the best random effect structure for LMM, we compared models with random intercepts only vs random intercepts and slopes, as well as different correlation matrices, and selected the most optimal model based on the lowest Bayesian Information Criterion value. The full models included PRS, sex, the top five PCs, time variable(s) and significant environmental factors (*p*<0.005 in the LMM or generalised estimating equation models for the outcome).

To evaluate if mechanisms differ by sex, we added the sex variable in interaction terms with either the pPRS, or an interaction between the pPRS and mean age of the visit. Associations with pPRSs were considered significant at *p*<0.01, applying a Bonferroni correction for the five independent pPRSs, while interactions with age were deemed significant if *p*<0.05.

### Persistent vs transient dysglycaemia

We examined whether the PRS can predict remission of dysglycaemia. For binary outcomes (prediabetes, type 2 diabetes, dysglycaemia, insulin resistance) with repeated assessments until visit F24, individuals were classified into three categories: Transient phenotype: those who lost the phenotype by age 18 or 24 years (last recorded visit); Persistent phenotype: those who retained the phenotype at this last recorded visit; Control category: those who never had the phenotype by age 18 or 24 years. Participants whose last recorded visit occurred before age 18 years were excluded. Multinomial logistic regression was applied using the *nnet* package using either the control or transient categories as reference, with analyses conducted only if each category had at least 100 individuals. AUC values were estimated using binomial logistic regression comparing each pair of categories. All models were adjusted for the top five PCs, sex, and age group at the last visit (18 or 24 years) to account for differences in follow-up duration.

Selection of environmental factors and control for collinearity followed the same approach as above. Only statistically significant environmental factors in univariate models (*p*<0.005) were included in a multivariate multinomial logistic regression (Model 3).

### Gene–environment interaction

To assess how environmental factors moderate genetic risk estimated by PRS, we included interaction terms between environmental factors and PRS in our models. For this purpose, PRS values were stratified into quintiles: low (Q1), intermediate (Q2–Q4) and high (Q5) genetic risk. Environmental factors were selected if both the PRS and the environmental variable were associated with the outcome at *p*<0.05 at the visit when the outcome was assessed in Model 3 of the cross-sectional analysis. All models were adjusted for age, sex and the top five PCs.

We evaluated both multiplicative and additive interactions. Multiplicative interactions, extracted directly from regression models, occur when the combined effect differs from the product of individual effects. Additive interactions, assessed using the relative excess risk due to interaction (RERI) in the *interactionR* package, occur when the combined effect exceeds the sum of individual effects [22].

Due to lower statistical power in interaction analyses, an interaction was considered significant if the PRS–environment term (high vs low genetic risk × environmental factor) had a *p*<0.05 and if the environmental factor was significantly associated with the outcome (*p*<0.05) in either group.

### Ethics approval and informed consent

Ethical approval for the study was obtained from the ALSPAC Ethics and Law Committee and the Local Research Ethics Committees. Informed consent for the use of data collected via questionnaires and clinics was obtained from participants following the recommendations of the ALSPAC Ethics and Law Committee at the time. Ethical approval for the study was obtained from the ALSPAC Law and Ethics committee and local research ethics committees (NHS Haydock REC: 10/H1010/70). Consent for biological samples has been collected in accordance with the Human Tissue Act (2004).

## Results

### Exposure and outcome variables in ALSPAC

Among the environmental factors, ten were measured in offspring in ALSPAC at many visits (ESM Table 1a), and eight were measured in mothers, either before or at different stages during pregnancy (ESM Table 1b).

A description of each outcome can be found in ESM Table 4. Overall, the prevalence of all binary outcomes increased across visits. For example, dysglycaemia had a prevalence of 2.76% at 7 years, and increased to 24.24% at 24 years.

### Association of genetic and environmental factors with glucose homeostasis outcomes

For our first objective, we compared models incorporating genetic factors, quantified by PRS, with those based solely on environmental factors. Results from cross-sectional analyses are detailed in ESM Figs 1–4 and ESM Table 5.

Specifically, we compared Model 2, including significant environmental factors, sex, PCs and age, to Model 3, which additionally incorporated PRS (ESM Tables 6, 7; ESM Figs 5, 6). For continuous outcomes in the full cohort, the R^2^ of Model 2 ranged from 2.11% (HOMA-IR at age 18 years) to 12.31% (fasting glucose at age 24 years). Incorporating PRS increased the R^2^ up to 43.86%, with the highest gain observed for the HbA_1c_ PRS in models for HbA_1c_ and for the fasting glucose PRS in models for fasting glucose at 15 years.

For binary outcomes, AUC values ranged from 0.55 (insulin resistant at age 15 years) to 0.76 (prediabetes at age 18 years). Adding PRS improved AUC values; for example, a fasting glucose PRS increased the AUC by 0.12 for predicting dysglycaemia and prediabetes at age 15 years, achieving a total AUC of 0.78. Similar findings were observed in sex-stratified analyses and when using the 85th percentile threshold.

### Longitudinal models

Several environmental factors were associated with outcomes across visits in Model 1 (ESM Tables 8, 9; ESM Fig. 7). Sex differences were notable. For insulin-related phenotypes, such as fasting insulin, HOMA-IR and insulin resistance, male individuals appeared to be at higher risk than female individuals.

Several PRSs, particularly for fasting glucose, were significantly associated with outcomes across visits (ESM Tables 8, 9; ESM Fig. 8). After adjusting for environmental factors, some associations were lost, although the effect size remained stable, suggesting reduced power rather than attenuation of genetic influence. Importantly, no significant temporal changes were observed for genetic effects, indicating that these effects remain stable throughout growth. This suggests that the fluctuations in associations observed in the cross-sectional analysis may be due to statistical power.

### Persistent vs transient dysglycaemia

The prevalence of dysglycaemia fluctuated over time, and most children classified with a binary outcome did not retain the phenotype into adulthood (ESM Table 4), as previously described in youth [13]. Among 958 children who met insulin resistance criteria at least once, only 340 remained insulin resistant at the final visit, consistent with physiological insulin resistance during puberty. Similar patterns were observed when using the alternative 85th percentile definition.

**Fig. 1:**
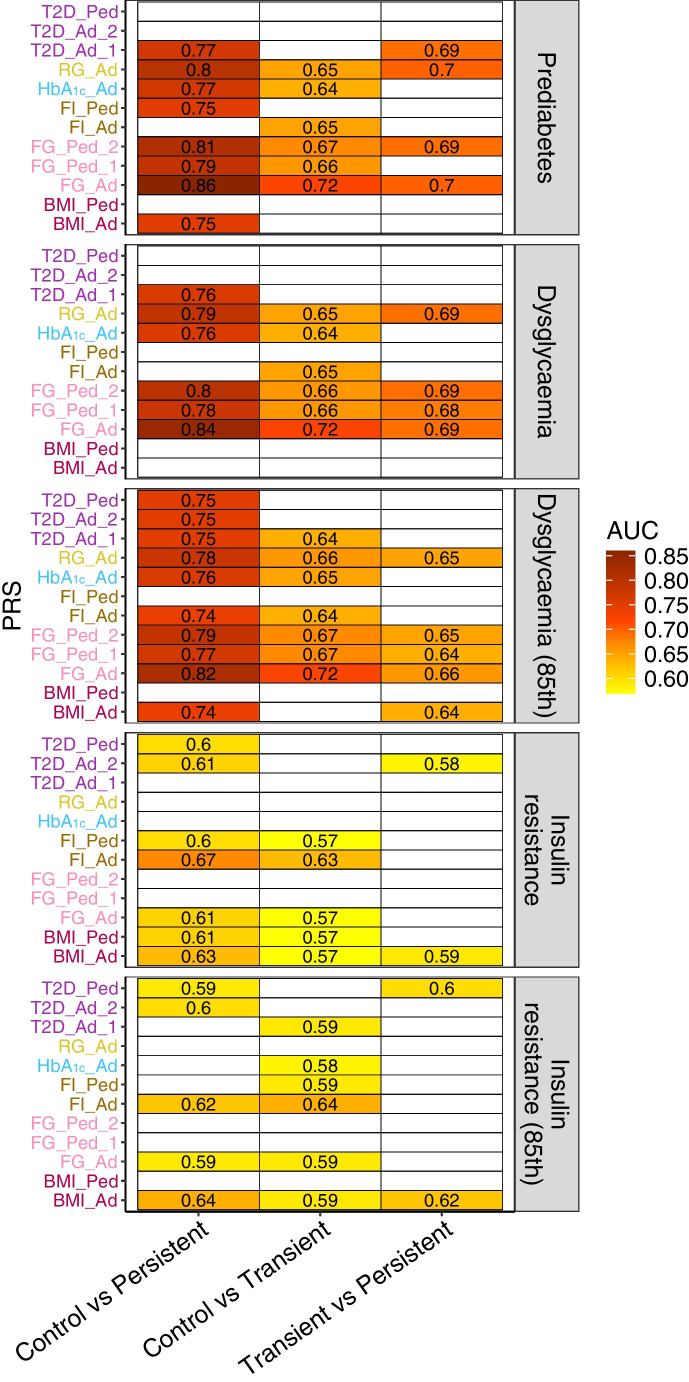
Heatmap displaying the AUC for persistent vs transient analyses within the entire cohort. Results are presented for significant comparisons (*p*<0.05 in multinomial models), adjusted for sex, top five PCs and age group of the last visit. PRS names are colour-coded based on the phenotypes for which they were generated. Ad, adult cohort; FG, fasting glucose; FI, fasting insulin; Ped, paediatric cohort; RG, random glucose; T2D, type 2 diabetes

We then evaluated whether PRSs could differentiate transient from persistent dysglycaemia (Fig. 1, ESM Table 10). Many PRSs distinguished transient from persistent prediabetes and dysglycaemia (based on both definitions), mostly those generated from random and fasting glucose levels, in both adults and children. However, AUC values remained modest, peaking at 0.70 [0.65–0.76] (*p*=6.90 × 10^−3^) for an adult random glucose PRS for prediabetes, indicating limited discriminatory performance of PRSs alone. For insulin resistance, BMI-related PRSs showed significant associations but with lower AUCs.

Adding environmental factors did not improve the AUC values (ESM Table 11), probably because of lower statistical power due to missing data.

### Gene–environment interaction

**Fig. 2:**
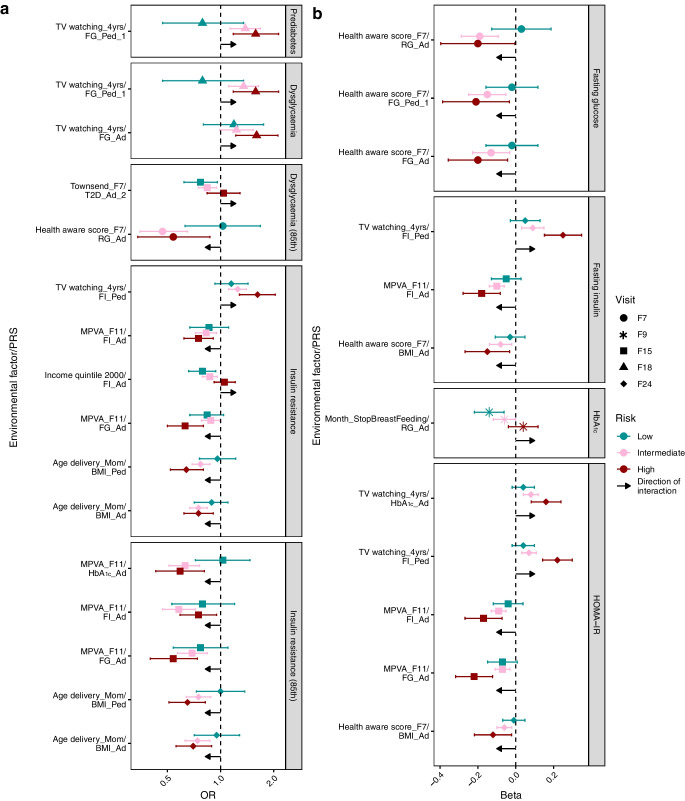
Significant interactions between environmental factors and PRS quintiles in the entire cohort for (**a**) binary outcomes and (**b**) continuous outcomes. Results depict associations in different genetic risk groups (coloured accordingly) and the additive interaction direction (RERI). Shape indicates the visit of the outcome. Visit or age information at the end of the environmental factor name indicates the visit or age at which the environmental factor was measured, except for Income quintile, which represents the year; Age delivery_Mom, which refers to the mother’s age at delivery; and Month_StopBreastFeeding*,* which represents the duration, in months, of the breastfeeding period. Ad, adult cohort; FG, fasting glucose; FI, fasting insulin; MVPA, moderate-to-vigorous physical activity; Ped, paediatric cohort; RG, random glucose; T2D, type 2 diabetes; TV, television; yrs, years

For the second objective, to assess whether environmental factors can modulate the impact of genetic susceptibility to dysglycaemia, we tested additive and multiplicative interactions between environmental factors and PRS quintiles (Fig. 2, ESM Table 12).

#### Binary outcomes

For binary outcomes, six environmental factors showed significant additive interactions, and four exhibited both additive and multiplicative interactions (Fig. 2a, ESM Table 12a). Notably, time spent watching TV at age 4 years and maternal income quintile showed positive interactions in models for dysglycaemia and prediabetes (TV), and for insulin resistance (income). The harmful effect of prolonged TV watching at age 4 years was amplified in individuals at high genetic risk, while the protective effect of high maternal income was weaker in this group, indicating a reduced buffering effect. Conversely, showing negative interaction, the beneficial impact of moderate-to-vigorous physical activity (MVPA) was stronger in those at high genetic risk, suggesting that physical activity could mitigate genetic susceptibility.

Since both advanced and young maternal age may impact child health [23, 24], and a significant negative interaction with maternal age was observed for insulin resistance, we stratified children based on maternal age (below or above the mean age). Notably, young maternal age at birth increased insulin resistance risk in individuals with high genetic risk, as the interaction was significant only in this group (b_BMI_Ad;InsulinResistance_F24;RERI_=−0.88 [−1.61, −0.16], *p*=0.02, *n*=781, maternal age range: 15 to 27 years), but not in the older age group (*p*_RERI_=0.08, *n*=1635).

In sex-stratified analyses, among the above interactions, only the MVPA interaction with a fasting glucose PRS in male individuals was significant for the binary outcome of insulin resistance.

#### Continuous outcomes

Among the continuous outcomes, five factors exhibited both additive and multiplicative interactions (Fig. 2b). Among these, two factors displayed a positive interaction. The first was prolonged breastfeeding, which showed a beneficial effect on HbA_1c_ levels at age 9 years in the low genetic risk group, but not in the high genetic risk group. The second was the harmful effect of prolonged TV watching on HOMA-IR and fasting insulin, which was more pronounced in individuals with high genetic risk. All other significant interactions were negative, reflecting amplified protective effects or decreased deleterious effects in individuals with high genetic risk. Notably, higher health awareness scores and greater MVPA levels were associated with stronger protective effects in high genetic risk groups, reinforcing the idea that certain lifestyle factors may counteract genetic predisposition to dysglycaemia

### Partitioned PRS

**Fig. 3:**
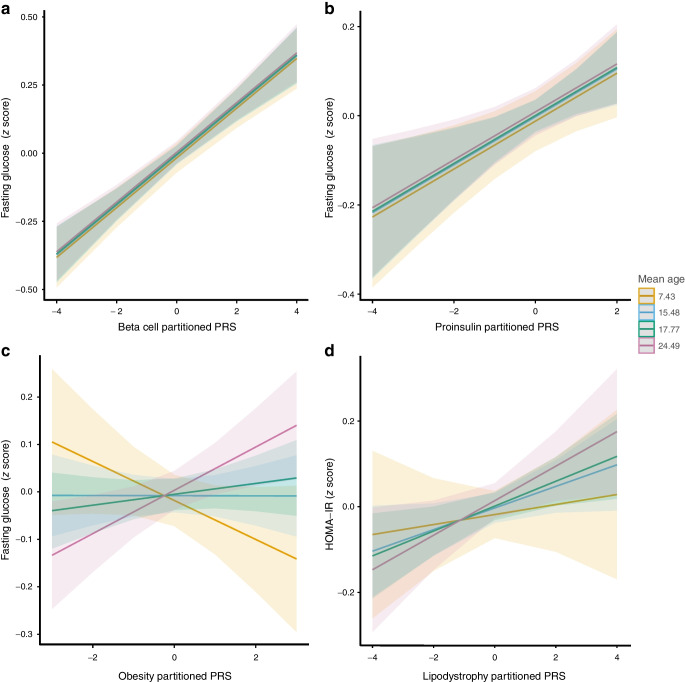
Linear mixed models for partitioned PRSs. The figure shows significant effects of pPRSs on outcomes across different visits (colour-coded). Each visit is named after the mean age of participants at that visit. Panels (**a**) and (**b**) present time trends without interaction with time for the (**a**) beta cell and (**b**) proinsulin pPRSs, both associated with fasting glucose levels (**a**) in the full cohort and (**b**) in male individuals. Panels (**c**) and (**d**) show time trends with a significant interaction with time for the (**c**) obesity and (**d**) lipodystrophy pPRSs, in the full cohort, associated with (**c**) fasting glucose and (**d**) HOMA-IR

For the third objective, we used pPRSs to investigate whether pathophysiological mechanisms evolve with age using longitudinal analyses (Fig. 3, ESM Table 13).

The pPRS associated with beta cell function was strongly associated with fasting glucose levels (b_FullCohort(BetaCell)_=0.09 ± 0.01, *p*=1.25 × 10^−13^, N_Ind_=4514 unique individuals, N_Obs_=8793 across visits) in both the entire cohort and sex-stratified analyses, with no significant variation over time (*p*>0.05), but with a weaker effect in female individuals (b_pPRS(Proinsulin)*sex_=−0.07 ± 0.02, *p*=0.005). Additionally, the pPRS for proinsulin was associated with fasting glucose in male individuals only (b_Male(Proinsulin)_=0.05 ± 0.02, *p*=0.003, N_Ind_=2,127, N_Obs_=4100), and this effect also remained stable across visits (*p*=0.69).

By contrast, pPRSs related to insulin resistance did not show significant associations with glycaemic traits across visits. However, significant interactions with time were identified. Notably, the pPRS for obesity exhibited a progressively stronger association with fasting glucose over time in the entire cohort (b_pPRS(Obesity)*time_=0.005 ± 0.002 SD by year, *p*=0.01, N_Ind_=4514, N_Obs_=8793) and in female-specific analyses. Similarly, the pPRS for lipodystrophy was associated with HOMA-IR and fasting insulin in male individuals, with increasing effects across visits. Moreover, the change in effect size over time differed by sex, with a smaller increase observed in female compared with male individuals for both the lipodystrophy and obesity-related pPRSs (b_pPRS(Lipodystrophy)*time*sex_=−0.009 ± 0.005, *p*=0.02), indicating that the annual effect increase is lower in female than in male individuals. Finally, none of the pPRSs was able to significantly differentiate persistent from transient dysglycaemia outcomes (ESM Table 13).

## Discussion

In this study, we examined how genes, environment and their interactions influence glucose homeostasis trajectories in a large European birth cohort followed into early adulthood. Our findings show that integrating PRSs with clinical, lifestyle, maternal and socio-demographic factors enhances dysglycaemia risk stratification throughout childhood and adolescence. While PRSs remained stable predictors over time, they were modestly performant to discriminate transient vs persistent dysglycaemia, suggesting a role for additional, likely non-genetic factors in glycaemic trajectories. We also found that more exercise, less TV watching, higher maternal income, higher health awareness and longer breastfeeding can partially offset genetic risk, highlighting the gene–environment interplay. Finally, using partitioned PRSs, we observed a shift in the underlying mechanisms of dysglycaemia over time and by sex: insulin production and secretion were more relevant in early life, whereas insulin resistance became increasingly influential with age, especially in male individuals.

### Contribution of the PRS to dysglycaemia risk prediction in youth

While several studies have constructed PRS for paediatric outcomes [25], most rely on SNPs identified through adult GWAS. However, genetic variants influencing traits in adults and children may differ [26], explaining the age-dependent predictive performance of these scores [25]. In our analysis, we found that adult-based PRS predicted paediatric outcomes as well as, or sometimes better, than those developed from paediatric cohorts. This likely reflects both the limited statistical power of smaller paediatric GWAS and the substantial genetic overlap between adult and paediatric type 2 diabetes [26, 27]. Our longitudinal results further support the consistency of the genetic determinants of glucose homeostasis across the lifespan, which is in line with prior findings [28]. Given the lifelong stability of the germline genotype, PRS may offer valuable tools for risk identification as early as at birth, allowing for targeted early prevention.

### Gene–environment interactions

UK Biobank studies have shown that a supportive environment can mitigate the impact of genetic predisposition to type 2 diabetes in adults [29]. Moreover, awareness and understanding of diabetes have been linked to better disease management [30], suggesting that education and health perception could also play a preventive role in youth. Consistent with prior findings [31, 32], our study shows that individual healthy behaviours, such as regular physical activity, limited screen time or greater health awareness, confer particular benefits to children at elevated genetic risk. These findings support the importance of promoting healthy habits in genetically vulnerable youth.

Beyond child-specific behaviours, maternal influences also shape early metabolic risk. Breastfeeding offers both short- and long-term benefits to offspring, including a lower risk of overweight and type 2 diabetes [33], while low income is a recognised risk factor for youth-onset type 2 diabetes [34]. Also, socioeconomically disadvantaged mothers often breastfeed for shorter durations [35]. In our cohort, shorter breastfeeding and lower maternal income were associated with increased dysglycaemia risk, independently of genetic risk. Although advanced maternal age (>35 years) is known to increase the risk of gestational complications and subsequent insulin resistance in offspring [23, 36], only 7% of mothers in our study met this criterion. Instead, we found that younger maternal age (15–27 years) was associated with greater offspring risk of insulin resistance. This age group often faces limited education and financial instability [24] and adopts less healthy lifestyles, and their infants are more likely to be born with low birthweight and experience adverse perinatal outcomes [24, 37], both of which are associated with later insulin resistance [38]. Most of these factors were considered in our analyses, suggesting that other mechanisms may also underlie this association. These findings reinforce the multifaceted impact of maternal age and environment on childhood metabolic health.

### Partitioned PRS

For our final objective, we found that the mechanisms driving dysglycaemia differ between childhood and adulthood. Insulin production and secretion appear to play a dominant role in earlier cases of dysglycaemia, while insulin resistance becomes more influential as youth approach adulthood. This shift might explain why type 2 diabetes progresses more rapidly in youth, who often experience faster beta cell decline and higher rates of metformin failure [38], contributing to the disease’s aggressive course during adolescence.

Previous research supports the idea of distinct biological pathways captured by pPRSs. For instance, in a paediatric cohort, the obesity-related pPRS was associated with higher BMI and fat mass, while pPRSs for lipodystrophy and beta cell were not associated [39]. In adults, the same study showed that lipodystrophy and beta cell pPRSs were associated with lower BMI, while the obesity pPRS remained linked to higher BMI, reinforcing the specificity of these genetic pathways across age groups.

Our results also highlight sex-specific differences in genetic susceptibility to dysglycaemia. While girls are more likely to develop type 2 diabetes in early childhood, in adulthood, men show higher prevalence and earlier onset [2, 3]. We found that pPRSs related to insulin production had stronger effects in male individuals across time, suggesting greater male vulnerability to beta cell dysfunction. Similarly, the influence of insulin resistance-related pPRSs increased with age, particularly in male individuals. However, the previously reported higher prevalence of dysglycaemia in girls during early childhood was not observed in ALSPAC, potentially due to its very low prevalence at 7 years of age.

In our analysis, we considered two definitions for dysglycaemia. The first definition followed the Diabetes Canada criteria, applying fixed thresholds across all ages. The second definition accounted for the normal age-related fluctuations in glucose and insulin levels, using age-specific cut-points at the 85th percentile, as previously implemented in ALSPAC [40, 41]. We followed the same approach by using two definitions for insulin resistance, based on standard HOMA-IR thresholds and age-specific 85th percentiles. Most results were consistent, supporting the robustness of our findings.

Together, these findings underscore the importance of accounting for both sex and age when interpreting genetic risk and support the development of tailored prevention strategies across growth stages.

Our study has several notable strengths. First, we leveraged data from a large, well-characterised longitudinal paediatric cohort, which allowed us to track glycaemic changes and genetic influences over time from childhood through adolescence. Second, rather than relying solely on the largest and most recent GWAS for type 2 diabetes, we drew from a broad set of GWAS encompassing diverse phenotypes related to type 2 diabetes in both adults and children. This comprehensive approach enhances our understanding of the multifaceted nature of the disease and enables the identification of individuals at risk through a wider range of metabolic phenotypes.

However, our study also has some limitations. The use of a food frequency questionnaire to assess dietary intake can introduce recall bias and misestimation [42], which may partly account for the unexpected direction of some observed associations. These findings may also reflect unmeasured confounding factors or compensatory behaviours, such as higher physical activity levels or overall better health in individuals reporting greater sugar consumption. A second limitation is that most of the PRSs used were derived from GWAS conducted in European populations, and our analyses were limited to a UK-based cohort. To address this, future work will focus on validating our findings in more diverse populations, including youth from Indigenous communities [43]. A third limitation is the relatively small number of dysglycaemia cases at the 7-years visit. Future studies combining data from multiple birth cohorts would improve statistical power and help clarify early life risk factors for dysglycaemia.

In summary, our study highlights the value of using PRSs and longitudinal paediatric data to uncover age- and sex-specific mechanisms underlying dysglycaemia. By integrating genetic risk to conventional risk factors and exploring their effects over time, including their interactions, we provide new insights into the pathogenesis of type 2 diabetes development from childhood onward. These findings underscore the need for early, personalised prevention strategies and support further research in ancestrally diverse paediatric populations.

## Supplementary Information

Below is the link to the electronic supplementary material.

ESM Figs (PDF 640 KB)

ESM Tables (XLSX 688 KB)

## Data Availability

ALSPAC data were available under the project number B-3723. Data on glycaemic traits generating PRSs from the GWAS cohort have been contributed by MAGIC investigators and have been downloaded from www.magicinvestigators.org. Sources for all PRSs are indicated in ESM Table 2.

## Abbreviations

ALSPAC: Avon Longitudinal Study of Parents and Children
GWAS: Genome-wide association studies
LMM: Linear mixed-effects models
MAGIC: Meta-Analyses of Glucose and Insulin-related traits Consortium
MVPA: Moderate-to-vigorous physical activity
PC: Principal component
pPRS: Partitioned polygenic risk score
PRS: Polygenic risk score
RERI: Relative excess risk due to interaction

## Software information & Supplementary tables

All references about software and R packages used across the manuscript can be found in ESM Table 14.

## Authors’ relationships and activities

MH is Chair of the Scientific Committee on Obesity Prevention at the Institut national de santé publique du Québec. She is also a member of the Advisory Group on the Management of Obesity in Children and Adolescents and the Québec Advisory Committee on Bariatric Medicine for the Québec Minister of Health and Social Services, and a member of the Professional Council of Diabète Québec. All other authors declare that there are no relationships or activities that might bias, or be perceived to bias, their contribution to this manuscript.
