# Supplementary material for: Nature vs nurture of glucose homeostasis trajectories in children from the ALSPAC study
Source: Diabetologia. 2026 Apr 15;69(7):1962–74. doi: 10.1007/s00125-026-06722-5 (PMC13236800; doi:10.1007/s00125-026-06722-5)
Supplement: Supplementary file 1 — ESM Figs (PDF 640 KB) [file 125_2026_6722_MOESM1_ESM.pdf]

ESM Fig.1

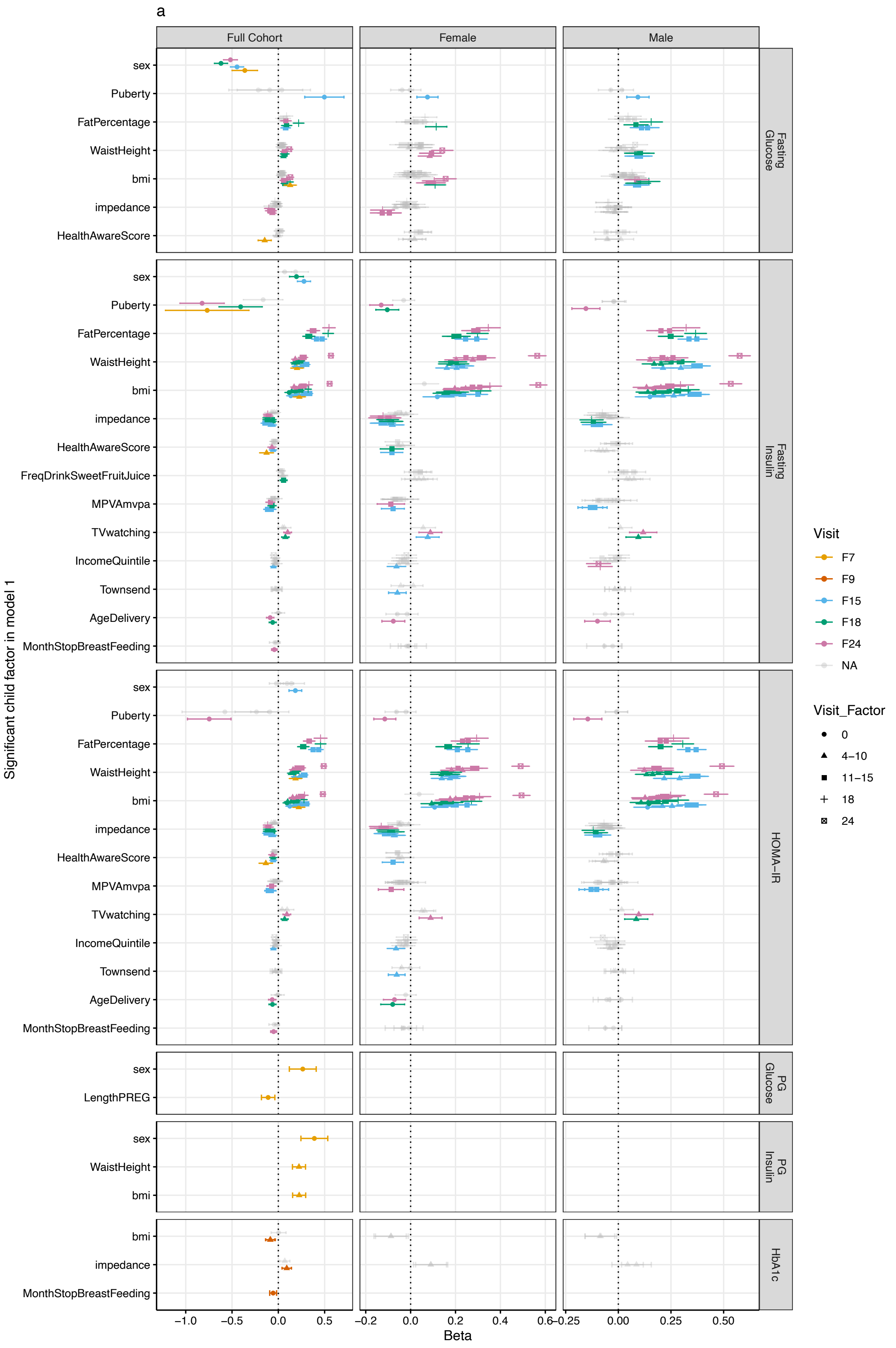

a
Full Cohort
Female
Male
Fasting Glucose
Fasting Insulin
HOMA−IR
PG Glucose
PG Insulin
HbA1c
sex
Puberty
FatPercentage
WaistHeight
bmi
impedance
HealthAwareScore
FreqDrinkSweetFruitJuice
MPVAmvpa
TVwatching
IncomeQuintile
Townsend
AgeDelivery
MonthStopBreastFeeding
LengthPREG
Significant child factor in model 1
Beta
−1.0
−0.5
0.0
0.5
−0.2
0.0
0.2
0.4
0.6
−0.25
0.00
0.25
0.50
Visit
F7
F9
F15
F18
F24
NA
Visit_Factor
0
4−10
11−15
18
24

ESM Fig.1

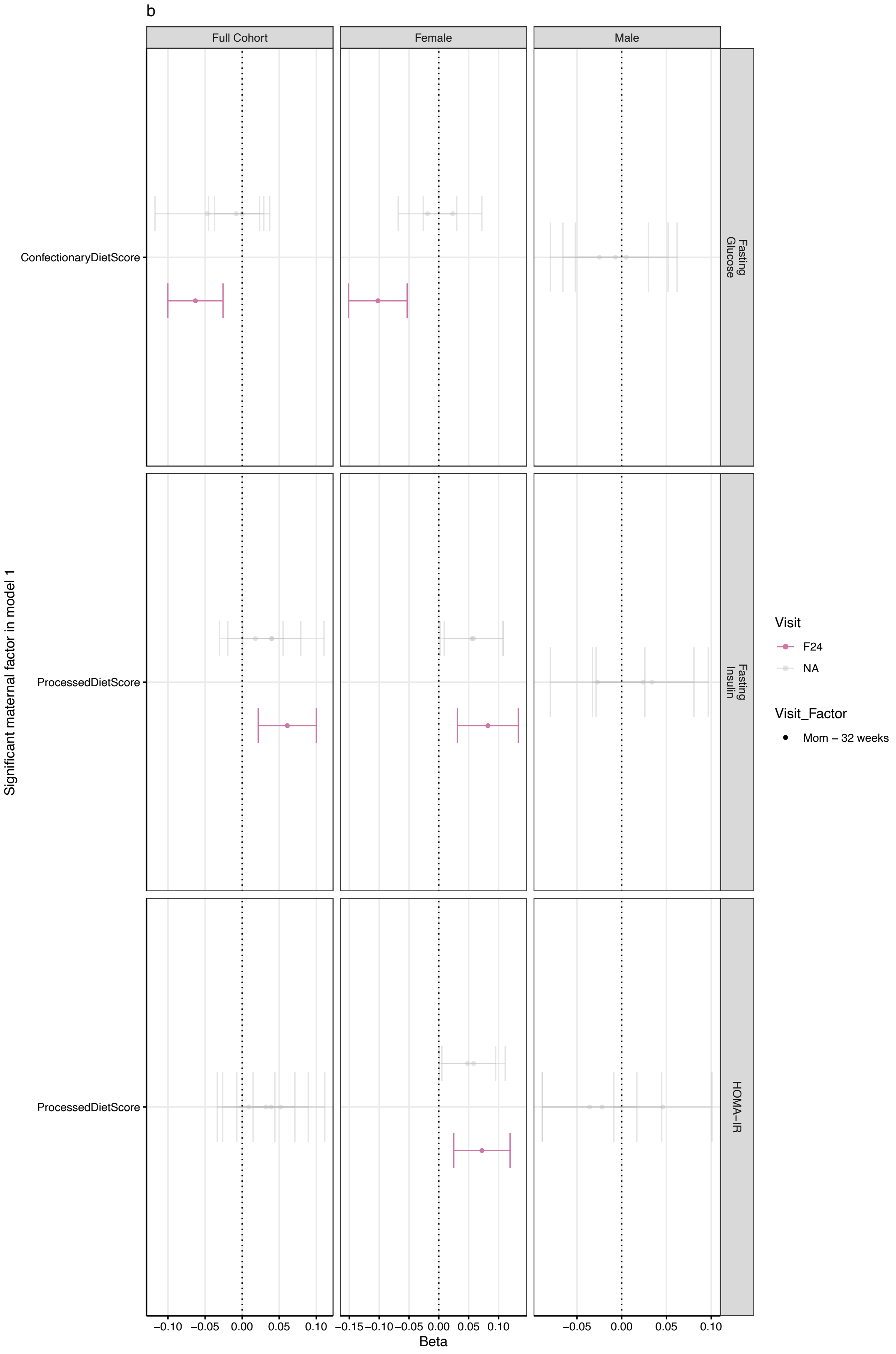

b
Full Cohort
Female
Male
Fasting Glucose
Fasting Insulin
HOMA–IR
ConfectionaryDietScore
ProcessedDietScore
ProcessedDietScore
Significant maternal factor in model 1
Beta
–0.10 –0.05 0.00 0.05 0.10
–0.15 –0.10 –0.05 0.00 0.05 0.10
–0.05 0.00 0.05 0.10
Visit
F24
NA
Visit_Factor
Mom – 32 weeks

ESM Fig. 1. Linear regression results from Model 1 evaluating environmental factors from both (a) child and (b) mother in the entire cohort and in sex-stratified analyses. Visits at outcome assessment are colour-coded only if the association is significant ($p<0.005$), while the timepoints at environmental factor measurements are indicated by different shapes.

Abbreviations: PG - 30 minute Post-Glucose test; IR - Insulin-resistance; bmi - Body Mass Index.

ESM Fig.2

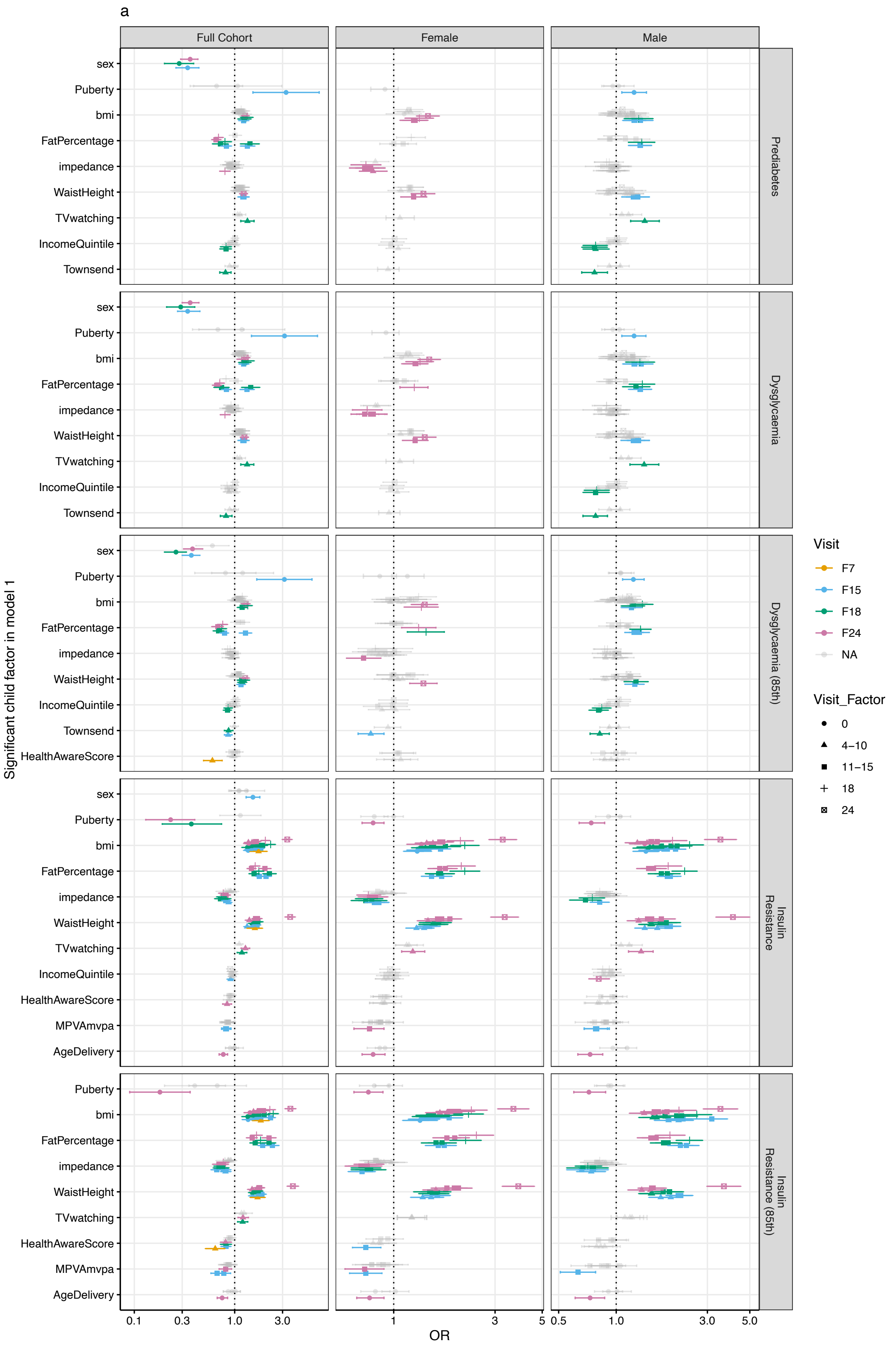

a
Full Cohort
Female
Male
Prediabetes
Dysglycaemia
Dysglycaemia (85th)
Insulin Resistance
Insulin Resistance (85th)
sex
Puberty
bmi
FatPercentage
impedance
WaistHeight
TVwatching
IncomeQuintile
Townsend
HealthAwareScore
MPVAmvpa
AgeDelivery
Significant child factor in model 1
OR
0.1
0.3
1.0
3.0
1
3
5
0.5
5.0
Visit
F7
F15
F18
F24
NA
Visit_Factor
0
4–10
11–15
18
24

ESM Fig.2

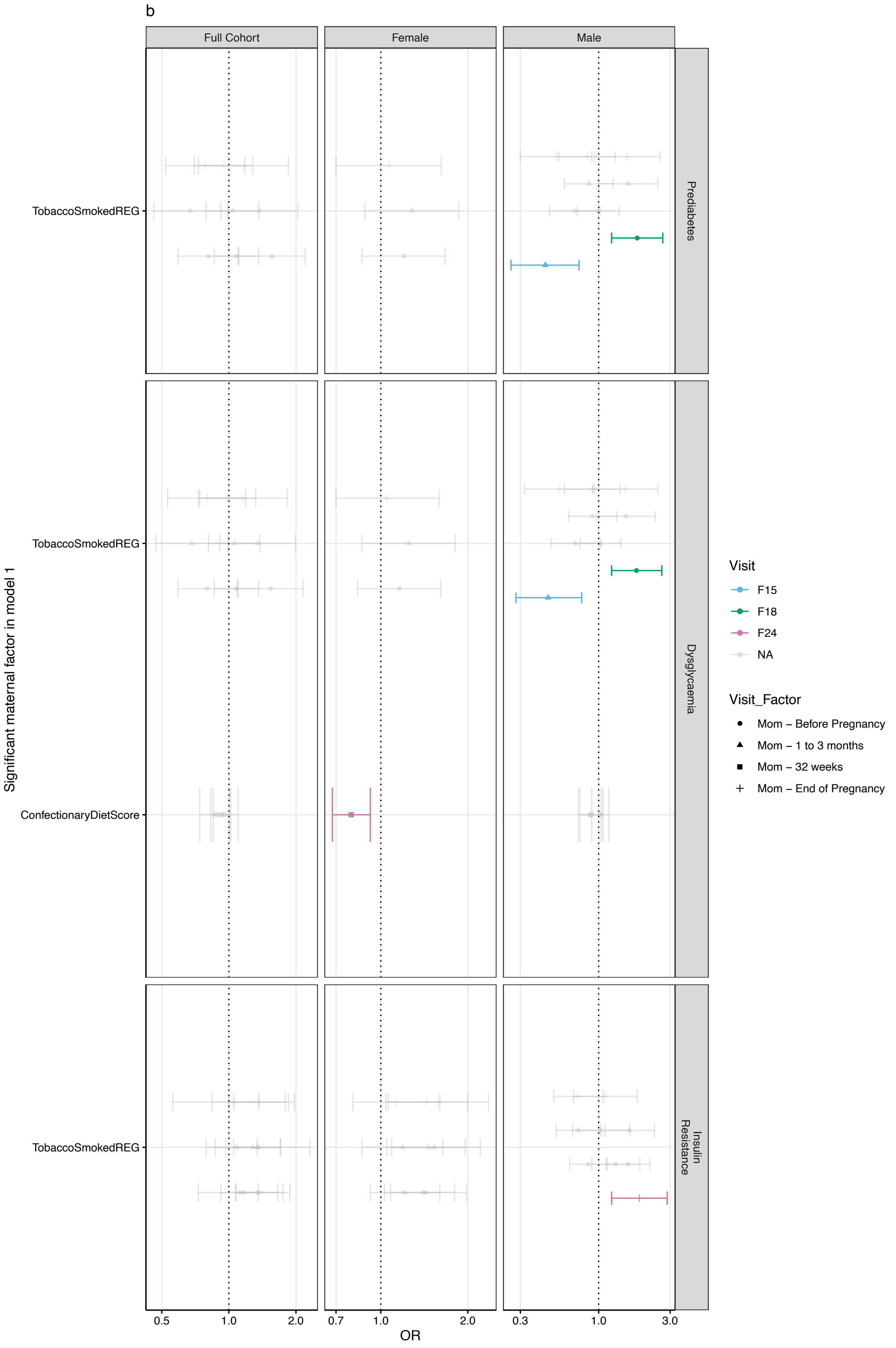

b
Full Cohort
Female
Male
Prediabetes
Dysglycaemia
Insulin Resistance
TobaccoSmokedREG
ConfectionaryDietScore
Significant maternal factor in model 1
0.5
1.0
2.0
0.7
1.0
2.0
0.3
1.0
3.0
OR
Visit
F15
F18
F24
NA
Visit_Factor
Mom – Before Pregnancy
Mom – 1 to 3 months
Mom – 32 weeks
Mom – End of Pregnancy

ESM Fig. 2. Logistic regression results from Model 1 evaluating environmental factors from both (a) child and (b) mother in the entire cohort and in sex-stratified analyses. Visits at outcome assessment are colour-coded only if the association is significant ($p<0.005$), while the timepoints at environmental factor measurements are indicated by different

shapes. Abbreviations: bmi - Body Mass Index.

ESM Fig. 3

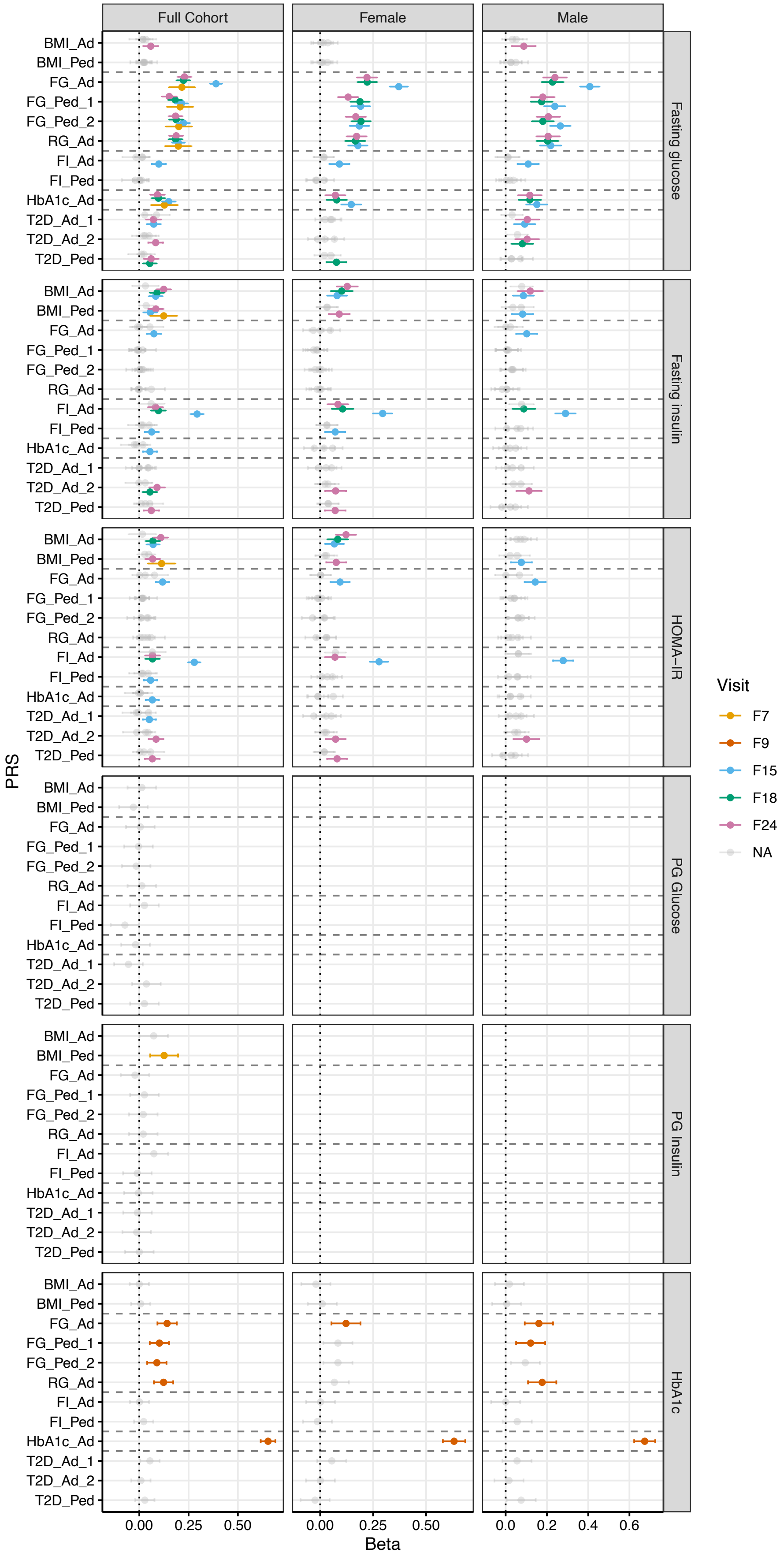

Full Cohort
Female
Male
Fasting glucose
Fasting insulin
HOMA–IR
PG Glucose
PG Insulin
HbA1c
BMI_Ad
BMI_Ped
FG_Ad
FG_Ped_1
FG_Ped_2
RG_Ad
FI_Ad
FI_Ped
HbA1c_Ad
T2D_Ad_1
T2D_Ad_2
T2D_Ped
PRS
0.00
0.25
0.50
0.0
0.2
0.4
0.6
Beta
Visit
F7
F9
F15
F18
F24
NA

ESM Fig. 3. Linear regression results from Model 1 evaluating PRS in the entire cohort and in sex-stratified analyses. Visits at outcome assessment are colour-coded only if the association is significant ($p<0.005$).

Abbreviations: PG - 30 minute Post-Glucose test; IR - Insulin-resistance; BMI - Body Mass Index; RG - Random Glucose; FI - Fasting Insulin; FG - Fasting Glucose; Ped - Paediatric cohort; Ad - Adult cohort.

ESM Fig.4

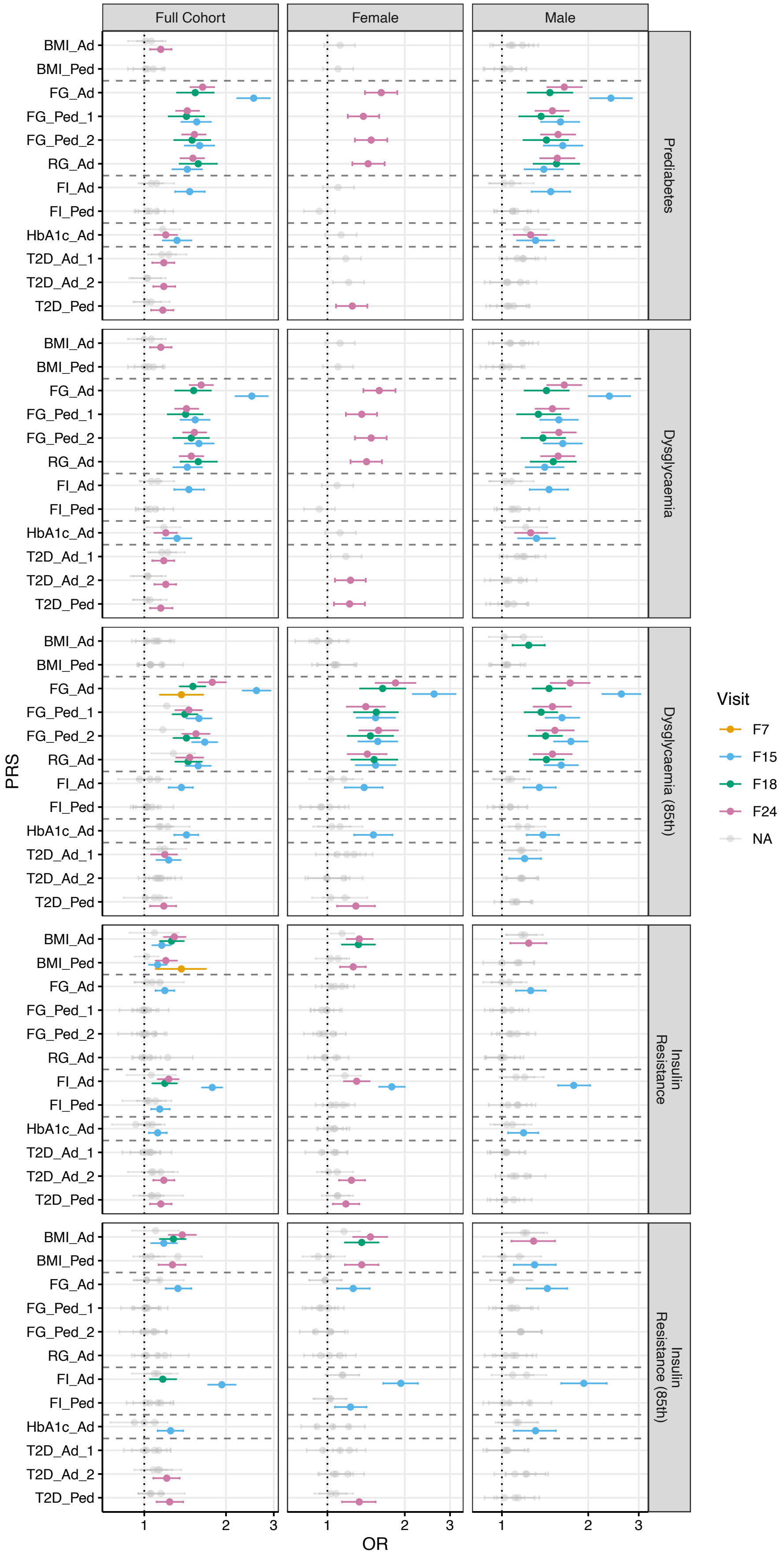

Full Cohort
Female
Male
Prediabetes
Dysglycaemia
Dysglycaemia (85th)
Insulin Resistance
Insulin Resistance (85th)
BMI_Ad
BMI_Ped
FG_Ad
FG_Ped_1
FG_Ped_2
RG_Ad
FI_Ad
FI_Ped
HbA1c_Ad
T2D_Ad_1
T2D_Ad_2
T2D_Ped
PRS
OR
1
2
3
Visit
F7
F15
F18
F24
NA

ESM Fig. 4. Logistic regression results from Model 1 evaluating PRS in the entire cohort and in sex-stratified analyses. Visits at outcome assessment are colour-coded only if the association is significant (p<0.005). Abbreviations: BMI - Body Mass Index; RG - Random Glucose; FI - Fasting Insulin; FG - Fasting Glucose; Ped - Paediatric cohort; Ad – Adult cohort.

ESM Fig.5

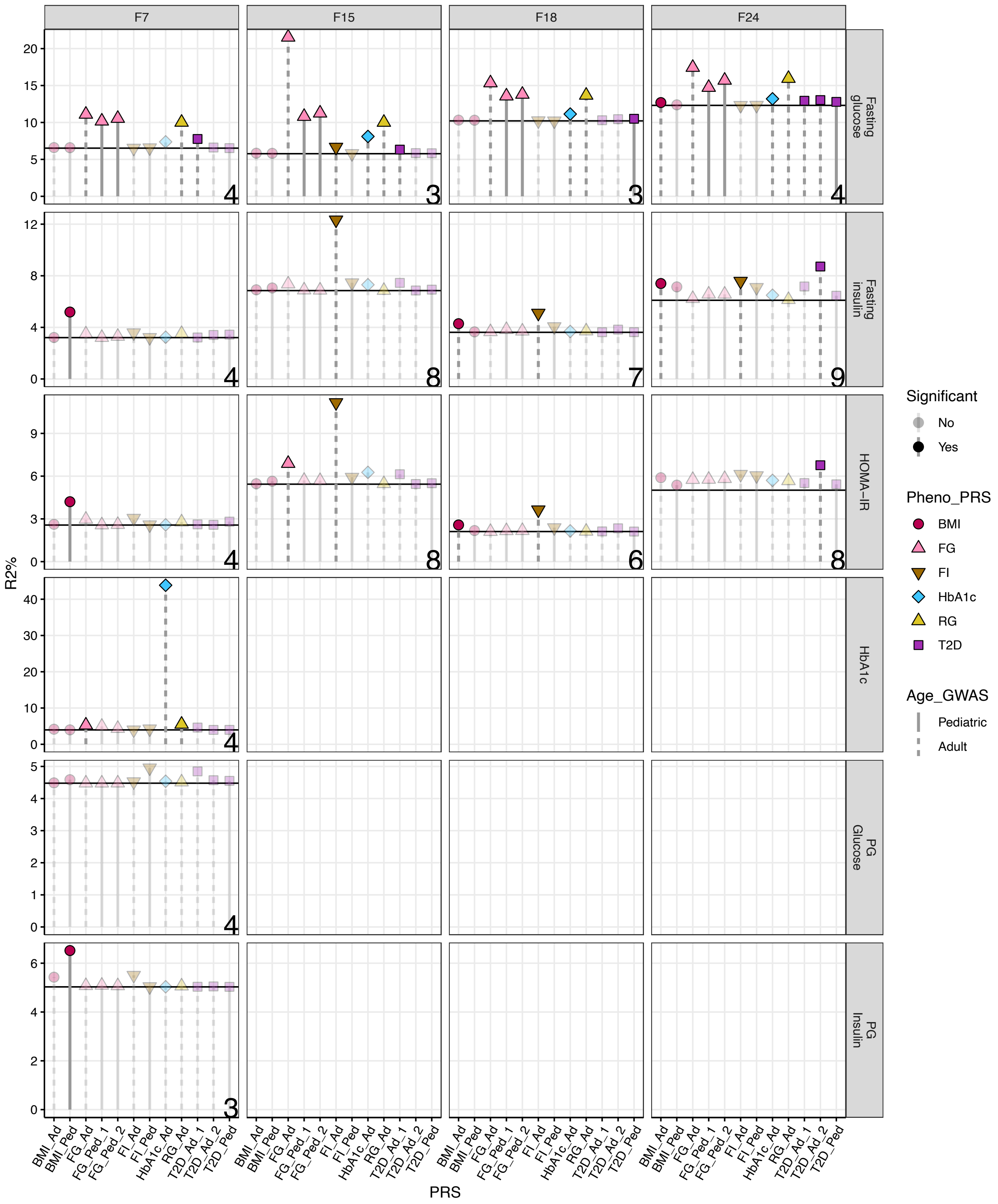

ESM Fig. 5. Lollipop chart displaying the explained variance (R2%) of each PRS across visits for each continuous phenotype. PRS are colour- and shape-coded based on the phenotype for which they were derived. Values are based on Model 3, while the variance explained by Model 2 is indicated by a horizontal reference line. Significant associations (p<0.005) are shown in full

opacity, whereas non-significant associations appear semi-transparent. The number of covariates - including sex, age, the top five PCs (counted as a single covariate), and significant non-collinear environmental factors - is noted in the bottom right corner of each subplot. Abbreviations: PG - 30 minute Post-Glucose test; IR - Insulin-resistance; BMI - Body Mass Index; RG - Random Glucose; FI - Fasting Insulin; FG - Fasting Glucose; Ped - Paediatric cohort; Ad - Adult cohort, T2D - Type 2 Diabetes.

ESM Fig. 6

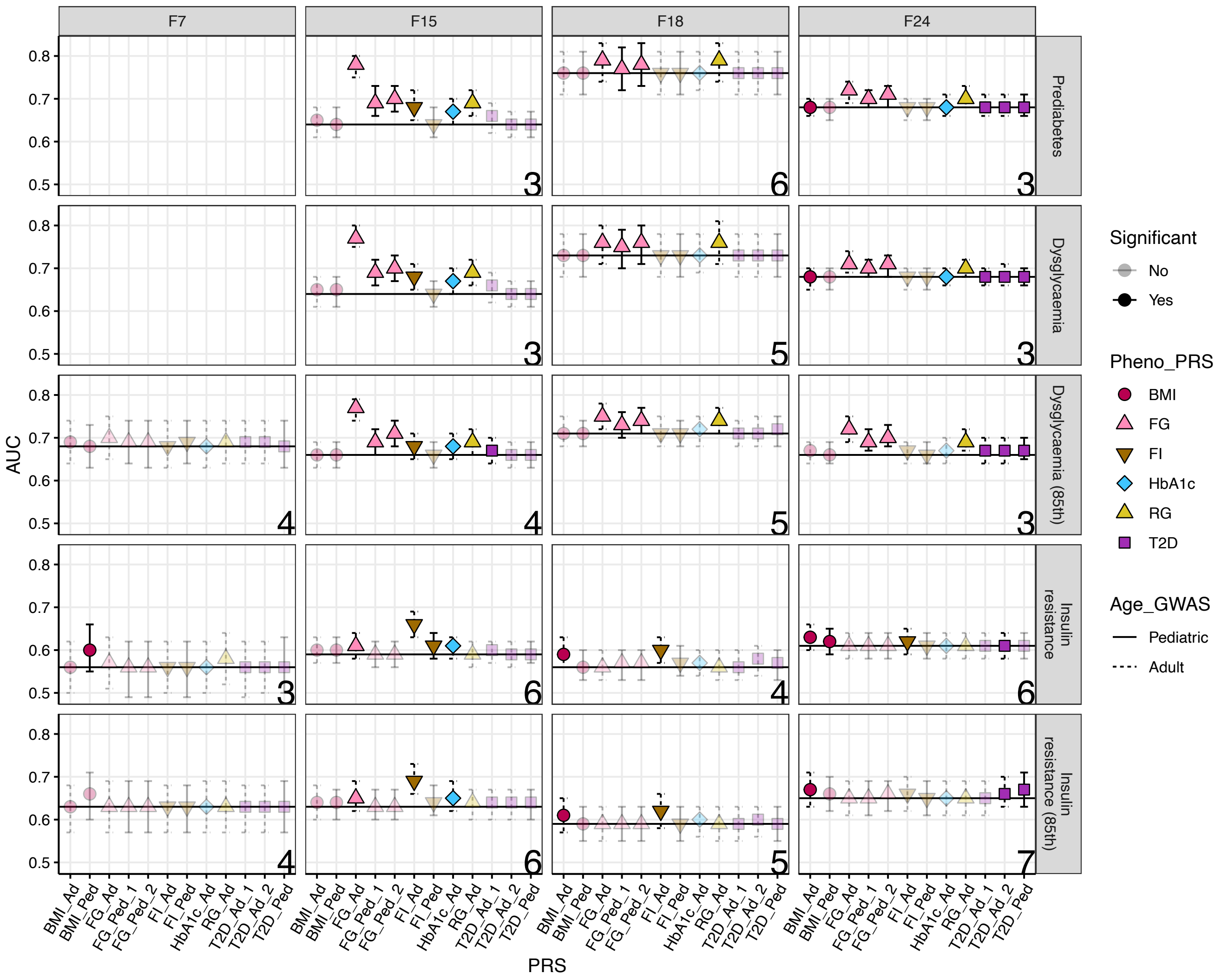

ESM Fig. 6. Plot displaying the area under the curve (AUC) and the standard error of each PRS across visits for each binary phenotype. PRS are colour- and shape-coded based on the phenotype for which they were derived. Values are based on Model 3, while the AUC of Model 2 is indicated by a horizontal reference line. Significant associations (p<0.005) are shown in full opacity, whereas non-significant associations appear semi-transparent. The number of covariates - including sex, age, the top five PCs (counted as a single covariate), and significant non-collinear environmental factors - is noted in the bottom right corner of each subplot. Abbreviations: BMI - Body Mass Index; RG - Random Glucose; FI - Fasting Insulin; FG - Fasting Glucose; Ped - Paediatric cohort; Ad - Adult cohort, T2D - Type 2 Diabetes.

ESM Fig.7a

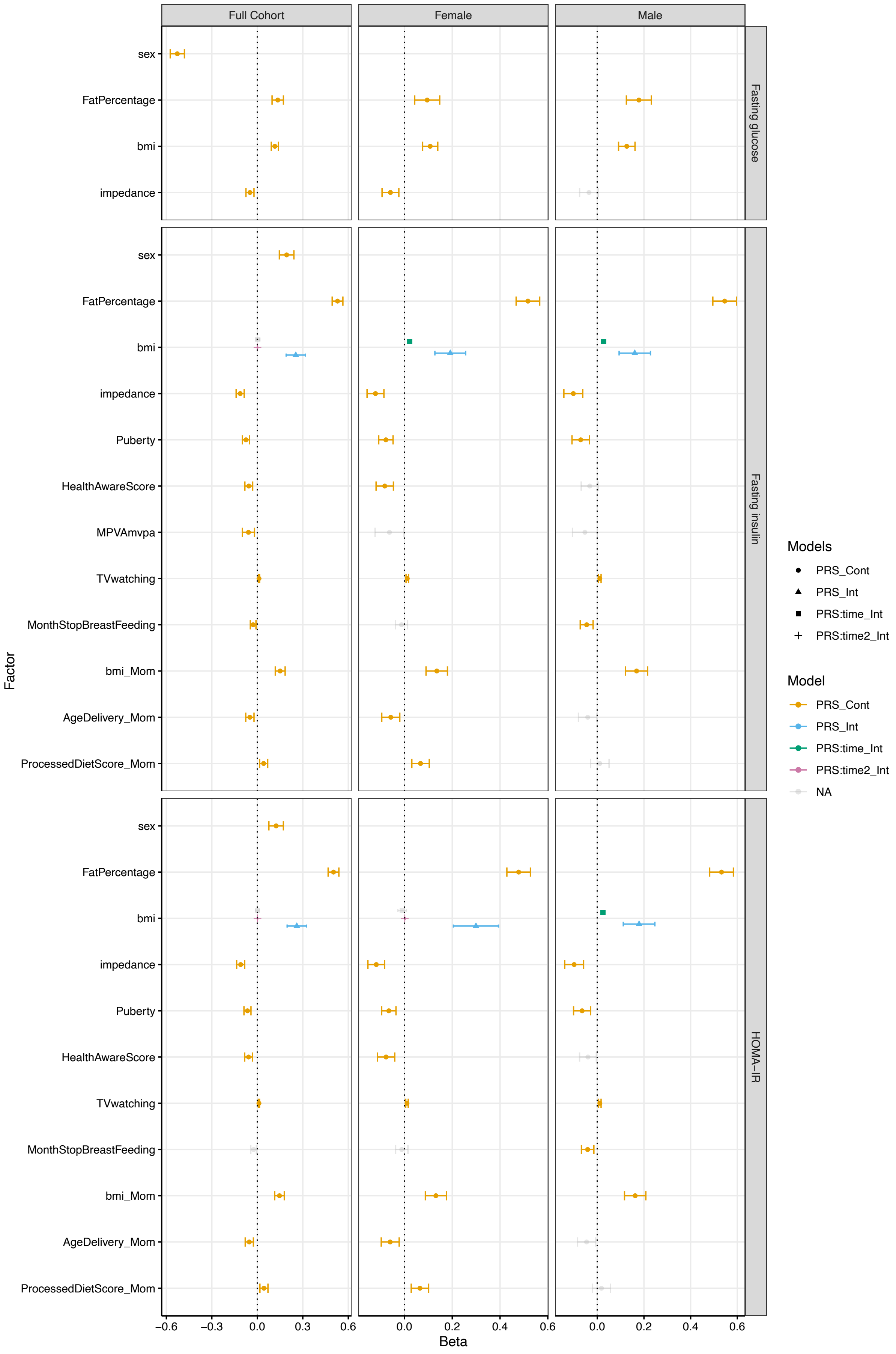

Full Cohort
Female
Male
Fasting glucose
Fasting insulin
HOMA−IR
sex
FatPercentage
bmi
impedance
Puberty
HealthAwareScore
MPVAmvpa
TVwatching
MonthStopBreastFeeding
bmi_Mom
AgeDelivery_Mom
ProcessedDietScore_Mom
Factor
Beta
−0.6
−0.3
0.0
0.3
0.6
0.2
0.4
Models
PRS_Cont
PRS_Int
PRS:time_Int
PRS:time2_Int
Model
PRS_Cont
PRS_Int
PRS:time_Int
PRS:time2_Int
NA

ESM Fig. 7b

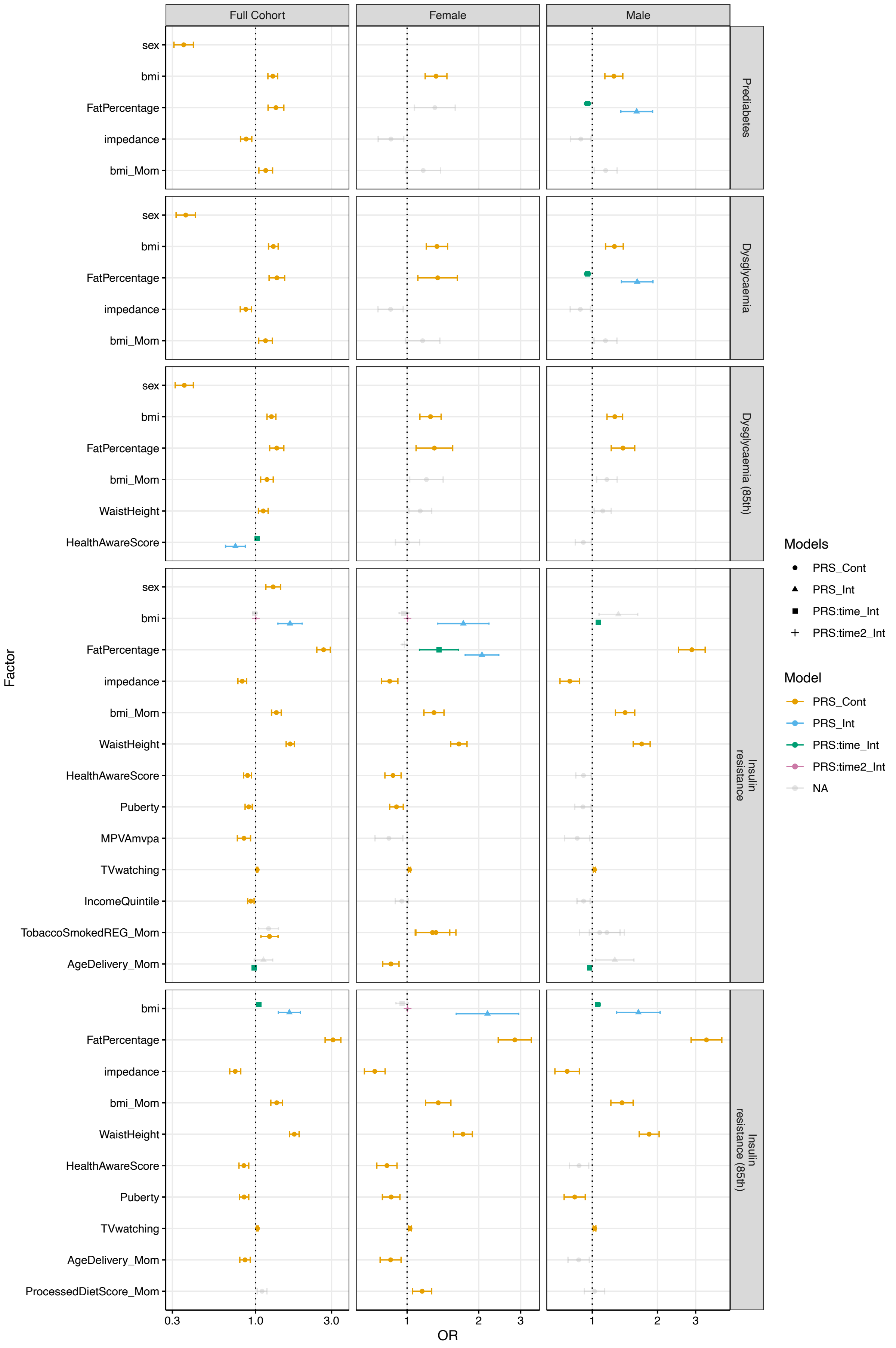

Full Cohort
Female
Male
Prediabetes
Dysglycaemia
Dysglycaemia (85th)
Insulin resistance
Insulin resistance (85th)
sex
bmi
FatPercentage
impedance
bmi_Mom
WaistHeight
HealthAwareScore
Puberty
MPVAmvpa
TVwatching
IncomeQuintile
TobaccoSmokedREG_Mom
AgeDelivery_Mom
ProcessedDietScore_Mom
Factor
OR
0.3
1.0
3.0
1
2
3
Models
PRS_Cont
PRS_Int
PRS:time_Int
PRS:time2_Int
Model
PRS_Cont
PRS_Int
PRS:time_Int
PRS:time2_Int
NA

ESM Fig. 7. Plots showing results from the linear mixed regression models for continuous phenotypes measured across multiple visits, across the entire cohort and in sex-stratified groups. Only significant environmental factors ($p<0.005$) are included. Results are derived from the best-fitting models, either with or without interaction with time. Most environmental factor values are scaled to one standard deviation; outcome values are also expressed in standard deviation units. Abbreviations: PRS_Cont - Effect of the PRS variable in the model without time interaction; PRS_Int - Effect of the PRS variable in the model with time interaction; PRS:time_Int - Effect of the PRS x time interaction term; PRS:time2_Int - Effect of the PRS x time2 interaction term; BMI - Body Mass Index.

ESM Fig.8a

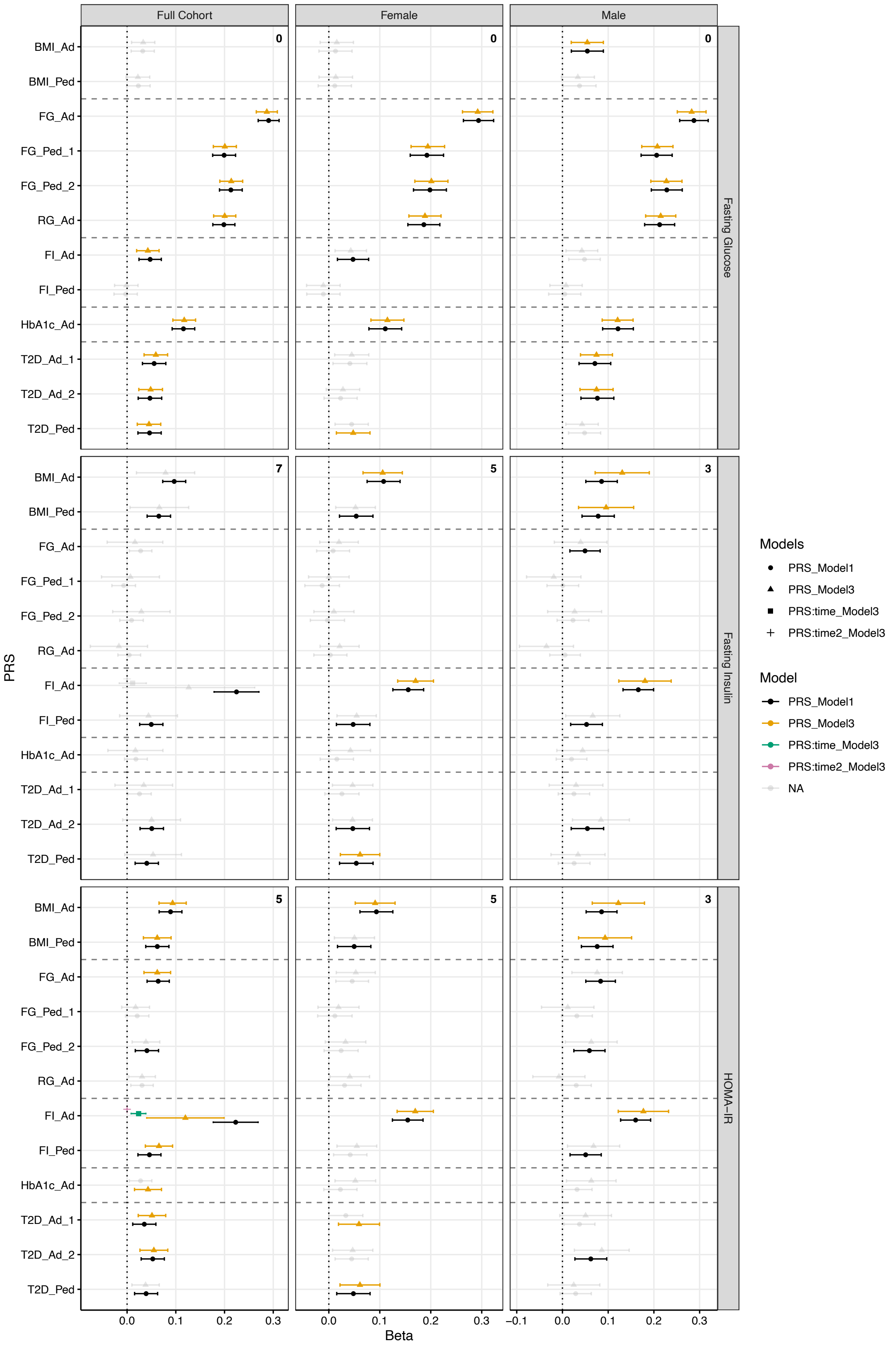

Full Cohort
Female
Male
Fasting Glucose
Fasting Insulin
HOMA−IR
BMI_Ad
BMI_Ped
FG_Ad
FG_Ped_1
FG_Ped_2
RG_Ad
FI_Ad
FI_Ped
HbA1c_Ad
T2D_Ad_1
T2D_Ad_2
T2D_Ped
PRS
Beta
0
7
5
3
Models
PRS_Model1
PRS_Model3
PRS:time_Model3
PRS:time2_Model3
Model
PRS_Model1
PRS_Model3
PRS:time_Model3
PRS:time2_Model3
NA

ESM Fig. 8b

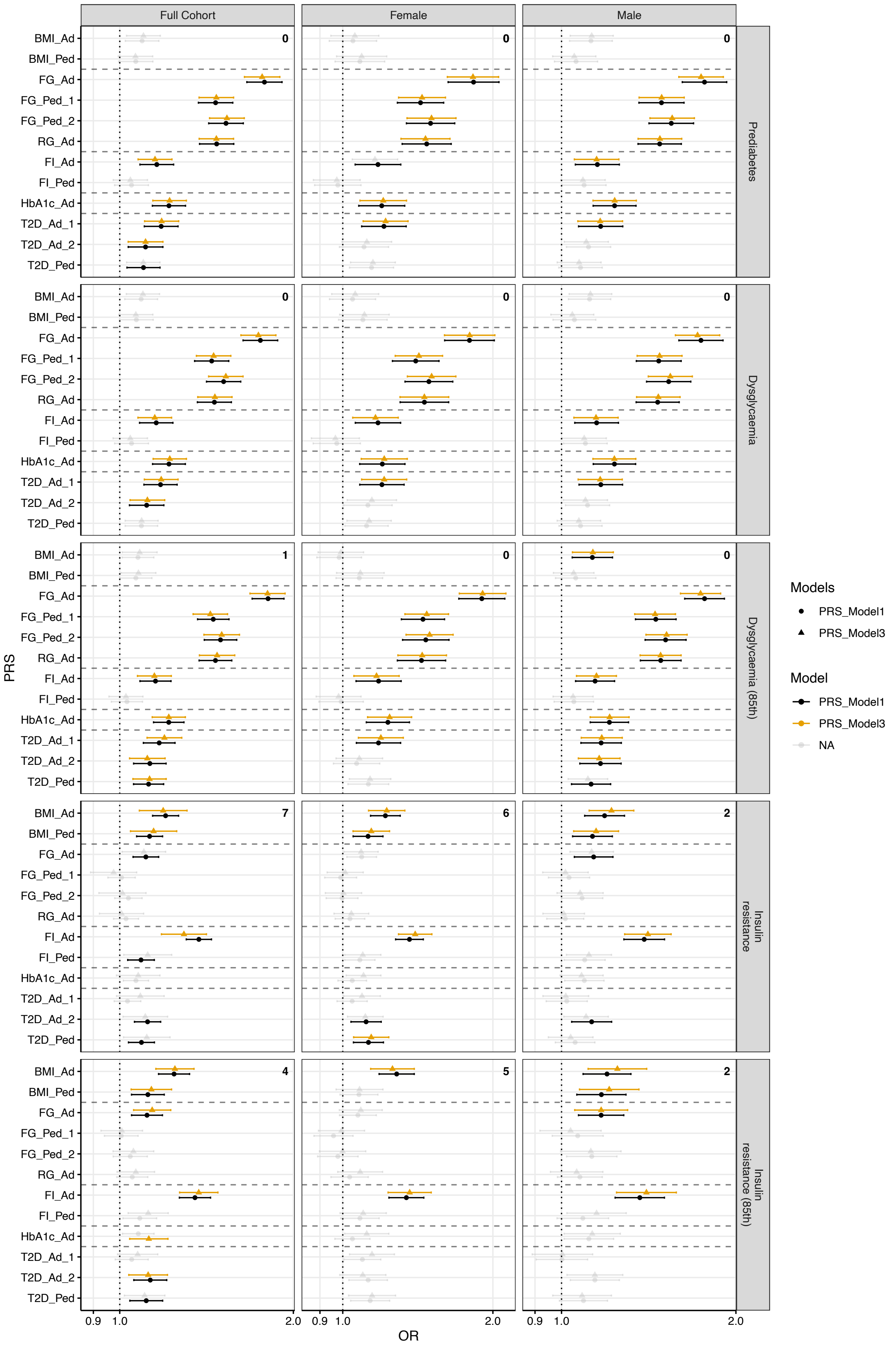

Full Cohort
Female
Male
Prediabetes
Dysglycaemia
Dysglycaemia (85th)
Insulin resistance
Insulin resistance (85th)
BMI_Ad
BMI_Ped
FG_Ad
FG_Ped_1
FG_Ped_2
RG_Ad
FI_Ad
FI_Ped
HbA1c_Ad
T2D_Ad_1
T2D_Ad_2
T2D_Ped
PRS
OR
0.9
1.0
2.0
0
1
7
6
2
4
5
Models
PRS_Model1
PRS_Model3
Model
PRS_Model1
PRS_Model3
NA

ESM Fig. 8. Plots presenting results from linear mixed regression models evaluating PRS effects on continuous phenotypes measured across multiple visits, shown for the entire cohort and in sex-stratified groups. PRS are shape-coded by model: Model 1 (without environmental factors) and the best-fitting Model 3 (including significant environmental factors), and if the effects from the PRS, PRS x time, or PRS x time2 variable. Significant results are color-coded according to the model displayed. The number of significant environmental factors (excluding sex, age, and PCs) is indicated in the top right corner of each subplot. Abbreviations: PRS_Cont - Effect of the PRS variable in the model without time interaction; PRS_Int - Effect of the PRS variable in the model with time interaction; PRS:time_Int - Effect of the PRS x time interaction term; PRS:time2_Int - Effect of the PRS x time2 interaction term; BMI - Body Mass Index; RG – Random Glucose; FI - Fasting Insulin; FG - Fasting Glucose; Ped - Pediatric cohort; Ad - Adult cohort, T2D - Type 2 Diabetes; Ad - Adult; Ped - Pediatric.
